# Oral famotidine versus placebo in non-hospitalised patients with COVID-19: a randomised, double-blind, data-intense, phase 2 clinical trial

**DOI:** 10.1136/gutjnl-2022-326952

**Published:** 2022-02-09

**Authors:** Christina M Brennan, Sandeep Nadella, Xiang Zhao, Richard J Dima, Nicole Jordan-Martin, Breanna R Demestichas, Sam O Kleeman, Miriam Ferrer, Eva Carlotta von Gablenz, Nicholas Mourikis, Michael E Rubin, Harsha Adnani, Hassal Lee, Taehoon Ha, Soma Prum, Cheryl B Schleicher, Sharon S Fox, Michael G Ryan, Christina Pili, Gary Goldberg, James M Crawford, Sara Goodwin, Xiaoyue Zhang, Jonathan B Preall, Ana S H Costa, Joseph Conigliaro, Joseph R Masci, Jie Yang, David A Tuveson, Kevin J Tracey, Tobias Janowitz

**Affiliations:** 1 Office of Clinical Research, Northwell Health, Lake Success, New York, USA; 2 Feinstein Institutes for Medical Research, Manhasset, New York, USA; 3 Department of Medicine, Donald and Barbara Zucker School of Medicine at Hofstra/Northwell, Hempstead, New York, USA; 4 Cold Spring Harbor Laboratory, Cold Spring Harbor, New York, USA; 5 New York City Helath + Hospitals Corporation, New York, New York, USA; 6 Medical Research Council Cancer Unit, University of Cambridge, Hutchison Research Centre, Cambridge, UK; 7 Medical School, University of Heidelberg, Heidelberg, Germany; 8 Northwell Health Cancer Institute, Northwell Health, New Hyde Park, New York, USA; 9 Department of Pathology and Laboratory Medicine, Donald and Barbara Zucker School of Medicine at Hofstra/Northwell, Hempstead, New York, USA; 10 Department of Obstetrics and Gynecology, Donald and Barbara Zucker School of Medicine at Hofstra/Northwell, Hempstead, New York, USA; 11 Biostatistical Consulting Core, School of Medicine, Stony Brook University, Stony Brook, New York, USA; 12 Department of Family, Population and Preventive Medicine, School of Medicine, Stony Brook University, Stony Brook, New York, USA; 13 Department of Neurosurgery, Department of Molecular Medicine, Donald and Barbara Zucker School of Medicine at Hofstra/Northwell, Hempstead, New York, USA

**Keywords:** COVID-19, clinical trials, inflammation, interferon

## Abstract

**Objective:**

We assessed whether famotidine improved inflammation and symptomatic recovery in outpatients with mild to moderate COVID-19.

**Design:**

Randomised, double-blind, placebo-controlled, fully remote, phase 2 clinical trial (NCT04724720) enrolling symptomatic unvaccinated adult outpatients with confirmed COVID-19 between January 2021 and April 2021 from two US centres. Patients self-administered 80 mg famotidine (n=28) or placebo (n=27) orally three times a day for 14 consecutive days. Endpoints were time to (primary) or rate of (secondary) symptom resolution, and resolution of inflammation (exploratory).

**Results:**

Of 55 patients in the intention-to-treat group (median age 35 years (IQR: 20); 35 women (64%); 18 African American (33%); 14 Hispanic (26%)), 52 (95%) completed the trial, submitting 1358 electronic symptom surveys. Time to symptom resolution was not statistically improved (p=0.4). Rate of symptom resolution was improved for patients taking famotidine (p<0.0001). Estimated 50% reduction of overall baseline symptom scores were achieved at 8.2 days (95% CI: 7 to 9.8 days) for famotidine and 11.4 days (95% CI: 10.3 to 12.6 days) for placebo treated patients. Differences were independent of patient sex, race or ethnicity. Five self-limiting adverse events occurred (famotidine, n=2 (40%); placebo, n=3 (60%)). On day 7, fewer patients on famotidine had detectable interferon alpha plasma levels (p=0.04). Plasma immunoglobulin type G levels to SARS-CoV-2 nucleocapsid core protein were similar between both arms.

**Conclusions:**

Famotidine was safe and well tolerated in outpatients with mild to moderate COVID-19. Famotidine led to earlier resolution of symptoms and inflammation without reducing anti-SARS-CoV-2 immunity. Additional randomised trials are required.

**Significance of this study**

**What is already known on this subject?**

- COVID-19 is caused by the SARS-CoV-2.
- Cytokine release drives inflammation and poor outcome in patients with COVID-19.
- Famotidine is a histamine 2 receptor antagonist that is globally used to reduce gastric reflux symptoms and treat gastric ulcers.
- In laboratory studies, famotidine reduced type-I interferon release from virally infected epithelial cells.
- Famotidine improved the outcome of patients with COVID-19 in some retrospective studies and a case series, but evidence from a clinical trial is lacking.

**What are the new findings?**

- In this randomised, double-blind and placebo-controlled clinical trial, oral famotidine was safe and well tolerated.
- Patient with mild to moderate symptoms from COVID-19 on famotidine experienced more rapid symptom resolution.
- Famotidine induced earlier resolution of type-I interferon levels in patients with COVID-19.

**Significance of this study**

**How might it impact on clinical practice in the foreseeable future?**

- High-density data studies with fewer patients may improve access to clinical trial participation from more institutions globally and require less resource.
- In the absence of strong alternatives, famotidine might be considered as a treatment for symptomatic outpatients with COVID-19.
- Additional clinical trials are needed and may leverage the knowledge generated by this study.

## Introduction

The search for safe, effective and affordable treatments for COVID-19 remains a global health priority. COVID-19 is pandemic, with an estimated 319 M cases and 5.5 M deaths worldwide to date.^1^ Restrictive public health measures in response to COVID-19 have led to unprecedented negative impacts on society.^2^

COVID-19 is caused by the SARS-CoV-2.^3^ On body entry, SARS-CoV-2 docks to the widely expressed ACE2^4 5^ and is internalised into the cell.^6 7^ Viral replication causes cell death and engages the immune system.^8^ Toll-like receptor 3 (TLR3) binds viral double-stranded RNA, leading to nuclear factor kappa-light-chain-enhancer of activated B cells directed transcriptional activation of type-I interferon and cytokine production.^9 10^ In most cases, the resulting inflammation and activation of the adaptive immune system leads to viral clearance, early resolution of inflammation and acquired immunity after a mild or even asymptomatic disease period.^11^ A significant minority of patients, however, experience moderate or severe COVID-19 caused by either suboptimal immune activation or non-abrogated inflammatory and immune overactivation, commonly referred to as a ‘cytokine storm’.^10^ This may explain why factors that predispose a patient to inflammation^12^ are risk factors for a poor outcome from COVID-19.^13^

COVID-19 preventions and treatments can be classified into the categories vaccination, anti-viral medication and host response modulator. Vaccinations prime the immune system, reduce disease severity and mortality and prevent spread of the disease.^14 15^ Unfortunately, their effectiveness may be compromised by viral variants, such as Delta or Omicron.^16 17^ Vaccine uptake is limited by non-concordance, cost, and supply and distribution hurdles.^18^ Similar factors may diminish the global impact of emerging anti-viral medications that have recently shown promising results in reducing hospitalisation and death of patients with risk factors for poor outcome.^19–21^ The sustained host inflammatory response has been modulated using anti-inflammatory medications and immune suppressants in hospitalised patients. For example, dexamethasone reduced mortality in hospitalised, oxygen-dependent patients with COVID-19.^22^ It has been shown to modulate interferon programming in patients with severe COVID-19.^23^ In outpatients with COVID-19, the selective serotonin reuptake inhibitor fluvoxamine reduced hospitalisation, but no mechanism is known.^24^

Famotidine is a widely available, safe, low-cost candidate medication for COVID-19. This selective histamine H2-receptor (H2R) antagonist reduced type-I interferon release from SARS-CoV-2-infected epithelial cells in a TLR3-dependent manner.^9^ Famotidine intake as an antacid has been associated with improved clinical outcome in several retrospective cohort studies of hospitalised patients,^25 26^ but some studies found no effect or negative associations.^27^ In a case series of unvaccinated outpatients with moderate COVID-19, oral famotidine at 80 mg three times a day was well tolerated and associated with rapid symptomatic and physiological improvement.^28^ Famotidine, as a result, has been frequently prescribed to non-hospitalised patients with COVID-19, without clinical trial data supporting biological or clinical efficacy.

We performed a randomised, double-blind, placebo-controlled, phase 2 clinical trial of oral famotidine (80 mg three times a day) and deeply profiled the enrolled diverse, non-hospitalised patients with mild to moderate symptoms from COVID-19. We devised a fully remote clinical trial strategy to reduce patient burden and exposure of the public and healthcare personnel. We aimed to assess the benefit of famotidine on resolution of symptoms and inflammation in patients with COVID-19.

## Methods

Extended information on methodology is provided in online supplemental file 1.

10.1136/gutjnl-2022-326952.supp1 Supplementary data

### Study design

**Figure 1:**
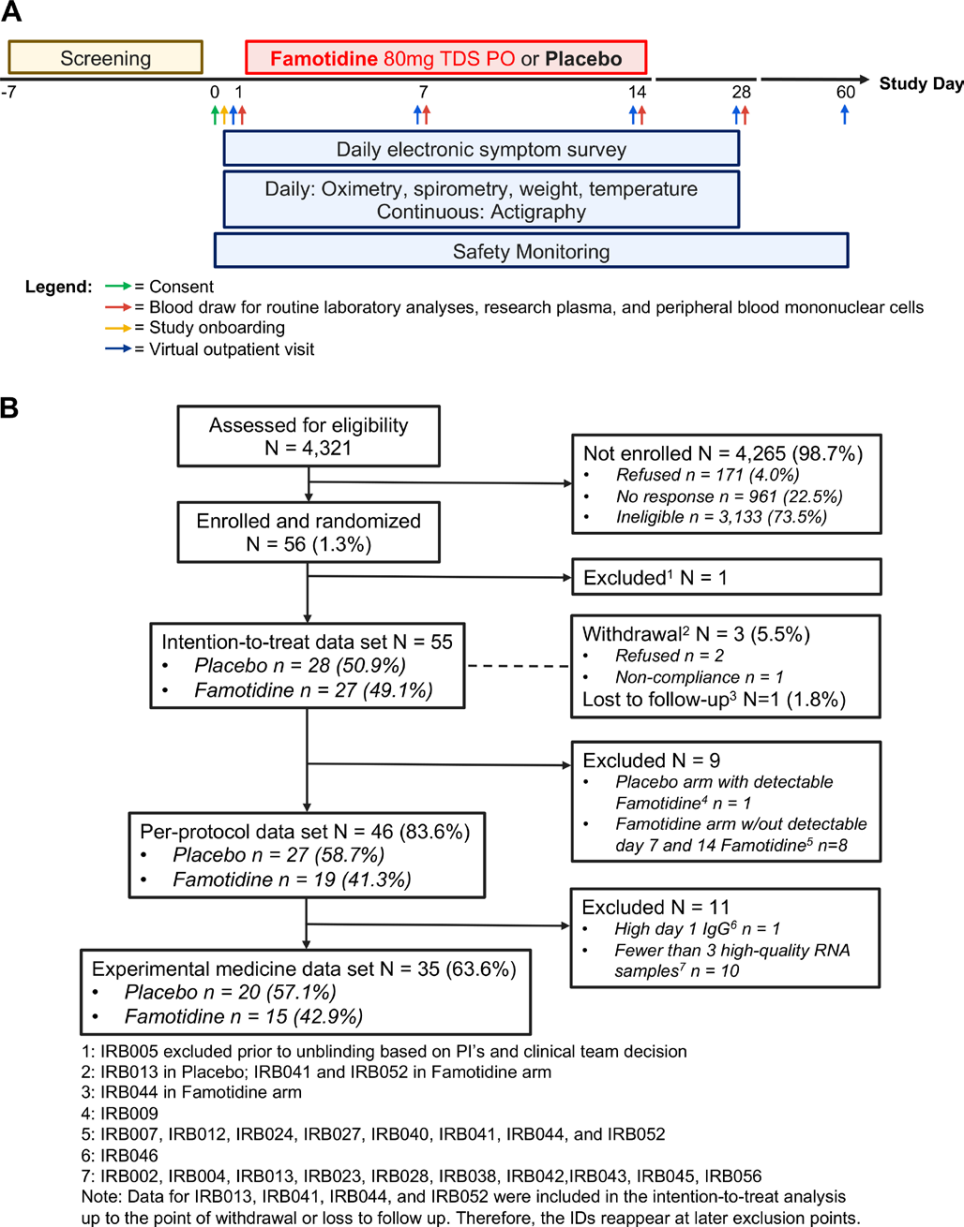
Trial overview. (A) The trial schematic and (B) the CONSORT diagram are displayed. IRB, Institutional Review Board; PO, per os, that is, taken by mouth; TDS, three times a day.

This randomised, double-blind, placebo-controlled, fully remote phase 2 clinical trial (figure 1A) was registered in the clinical trials database of the National Institutes of Health (NCT04724720). Following consent, patients were supplied a dedicated electronic tablet device to electronically submit for a period of up to 28 days scores for 17 symptoms: lack of energy, shortness of breath, cough, headache, loss of smell or taste, loss of appetite, difficulty of breathing, diarrhoea, sore throat, muscle pain, hoarse voice, runny/stuffy nose, chest tightness, abdominal pain, nausea, dizziness and eye discomfort. Each symptom was scored by the patient on an ordinal scale: 0=none, 1=mild, 2=moderate, 3=severe. Discontinuation of daily symptom score was permitted when sustained symptom resolution had occurred. Patients also obtained and submitted daily oxygen saturation, peak flow spirometry, body weight and body temperature readings using electronic devices at home for up to 28 days.

From the day after consent (day 1), a period of 14 days of study medication intake was followed by 14 days of continued daily monitoring and a final telephone safety consultation and symptom review on day 60.

Blood samples and nasopharyngeal swabs were collected by a mobile phlebotomy service at the patient’s residence on days 1, 7, 14 and 28. Remote blood draws on day 1 were timed prior to the patient’s first dose of study medication. These blood draws were used to isolate peripheral blood mononuclear cells (PBMCs), to analyse the complete blood counts and to isolate plasma for all of the biochemical, famotidine and interferon α analyses.

### Patient recruitment and patients

We screened and enrolled patients from two US sites: Northwell Health (NH) and New York City Health and Hospitals Corporation (New York Health+Hospitals or NYCHHC). Candidates were identified from laboratory lists, emergency department discharge lists (NH) or from COVID-19 isolation hotel admissions (NYCHHC) and contacted by screening team members by telephone or in person at the hotels by the nursing staff (online supplemental figure S1A). Enrolled patients were at least 18 years old and had a laboratory-confirmed COVID-19 diagnosis (RT-PCR) less than 72 hours prior to randomisation, a minimum of three symptoms of moderate severity for 1–7 days and the ability to use electronic devices. Exclusion criteria were a history of anti-SARS-CoV-2 vaccination, receipt of antibody or steroid treatment for COVID-19, known autoimmune disease (ie, diagnosed with rheumatoid arthritis, systemic lupus erythematosus or similar), prolonged QTc interval or glomerular filtration rate below 60 mL/min/1.73 m^2^, suspected dysphagia and famotidine intake at the time of screening. Pregnant patients were eligible for enrollment.

Central randomisation, data storage, trial coordination, pharmacovigilance, patient follow-up and shipment of trial monitoring kits were performed by the Northwell Health Office of Clinical Research. All aspects of the study were conducted in accordance with the Declaration of Helsinki, including all of its relevant amendments, the guidelines for Good Clinical Practice of the International Conference on Harmonization, and all relevant New York state and US laws and directives. An independent monitor reviewed all study data. An independent Data Safety and Monitoring Board (DSMB) monitored recruitment, subject safety and outcome.

### Data safety

All patients received a unique study ID, IRB001 to IRB056, and patient-reported data were submitted using the study ID by secure data link, in a Health Insurance Portability and Accountability Act of 1996 (HIPAA)-compliant manner, to a secure data storage portal. Data were then combined with the full REDCap trial database prior to analysis.

### Randomisation and masking

We randomly assigned patients in a 1:1 ratio to either placebo or famotidine treatment groups using central block randomisation generated by the Northwell Office of Clinical Research. Stratification was by patient sex and study site. Trial participants, investigators and trial staff remained blinded for the entirety of the study duration and clinical data analysis.

### Treatment, concordance and toxicity assessment

Size #000 opaque white capsules (Gelatin-free Capsugel) either filled with microcrystalline cellulose (Avicel, FMC Corporation, Madison, Wisconsin, USA) or famotidine 80 mg (20 mg tablets×4; overencapsulated) were manufactured by Alchem Laboratories in concordance with current Good Manufacturing Practice regulations (online supplemental figure S1B). Patients self-administered three capsules a day approximately 8 hours apart for 14 days from day 1.

To assess toxicity, patients were reviewed by telehealth consultation, and complete blood count, serum liver chemistries and serum creatinine with estimated glomerular filtration rate were monitored. All results and adverse events were reviewed and acted on by the blinded principal investigator and blinded clinical team. The blinded DSMB reviewed all adverse events, and no events that required unblinding were encountered.

### Statistical analysis and missing data

Data were collected from the REDCap database downloaded on 30 August 2021 and VitalCare Symptom Surveys on 12 May 2021. All symptom-related and general inflammatory endpoints were analysed using both intention-to-treat (ITT) analysis and per-protocol (PP) analysis of the subgroup of patients who had confirmed famotidine intake by plasma analysis. Statistical analysis was performed using SAS V.9.4 (SAS Institute), and the significance level was set at 0.05. We believed data was missing at random, and did not impute missing data.

#### Endpoints

The endpoints were developed in close collaboration with the Food and Drug Administration. The strength and weaknesses of using fewer data points and comparing a single endpoint that captures sustained clinical recovery or of using all survey supplied daily total symptom scores and comparing modelled change of symptoms over time were considered. A shared decision was made to capture both these symptom resolution endpoints as primary and first secondary endpoint, reflecting that this phase 2 trial was in part designed to identify the strongest endpoint for future studies.

#### Cumulative incidence of symptom resolution at day 28

Time to symptom resolution was defined as days from treatment start to either the first-time achieving symptom resolution or the last follow-up, up to day 28, whichever occurred first. Symptom resolution was defined as the first time that the total symptom score was ≤3, and no individual symptom score was >1 for two consecutive days. The famotidine and placebo arm were compared using stratified log-rank test.^29^ As all the measurements were monitored through one site, Northwell Health, only sex was stratified in model instead of both sex and study site.

#### Relative change in symptom score

The decreasing rate of symptom resolution from day 0 to day 28 was assessed using a mixed random effect model assuming a linear change over time in ln(score+1) which was applied to overall symptom score to meet the normality model assumption. Factors adjusted in the model included time (Day in Study), treatment arm and sex. The covariance structure considered to model the correlation among longitudinal measurements from the same patient was Toeplitz. The selection was based on convergence status and Arkaike Information Criteria. Other structure considered included compound symmetry (CS), heterogeneous CS, first-order autoregressive (AR(1)), heterogeneous AR(1) and unstructured. Two-way interaction term between treatment arm and time was used to model the different changing rate in each treatment arm. To explore if difference in the changing rate between two study arms was similar between sex, race and ethnicity groups, corresponding three-way interaction terms were further examined in the linear mixed effect model.

#### Individual symptom resolution

Cumulative incidence of resolution (symptom score decreased to ≤1 for two consecutive days) of each individual symptom that are >1 at baseline (day 0) was estimated for days 7, 14, 21 and 28. The symptom diarrhoea was not included in this analysis because it had low baseline prevalence and all patients were asymptomatic before day 7. Analysis method was correspondent to the analysis of primary endpoint.

#### Relative change in C reactive protein (CRP) and ferritin level

CRP and ferritin levels were measured on days 1, 7, 14 and 28. Values under detection limit were treated as missing. Linear mixed effect models were used to compare the levels at different time points for each treatment arm. Log-transformation was applied, that is, ln(level), to meet the normality assumption. Factors adjusted in models included time (day in study), treatment arm and gender. Time was treated as a discrete variable. The interaction term between treatment arm and time was used to estimate ferritin relative change over time within each treatment group. Based on the similar strategy, the selected covariance structure in the models was CS.

#### Power calculation

The power calculation was based on an assumption of cumulative incidences of symptom resolution at day 28 in the treatment arm and placebo arm of 80% and 50%, respectively. Based on this assumption, 84 patients would have achieved 92.34% power to detect such a difference using a two-sided log-rank test with type-I error controlled at 0.05.

### Plasma famotidine level

Liquid chromatography-mass spectrometry with isotope-labelled internal standardisation for famotidine was used to determine calculated plasma famotidine concentrations.

### RNA isolation from PBMCs

RNA was isolated from PBMCs using the PureLink RNA Mini Kit (Cat. No. 12183025, Thermo Fisher Scientific, Waltham, Massachusetts, USA).

### RNA sequencing analysis

RNA sequencing libraries were generated by poly(A) capture prior to reverse transcription into reverse strand-specific cDNA libraries. Sequencing (100 bp, paired-end) was performed on a NextSeq 2000 machine with a P3 kit (Illumina, San Diego, California, USA). Read alignment and quantification was implemented using the nf-core/rnaseq pipeline V3.0.^30^ Briefly, reads were aligned to the GRCh38 reference using GENCODE v36 reference annotations with STAR,^31^ and RSEM^32^ was used for gene-level quantification. Gene count matrix normalisation was implemented in edgeR to generate Trimmed Mean of M-values (TMM)-normalised transcripts per million expression values, which are suitable for between-sample and within-samples analyses.^33^ Single-sample gene set enrichment analysis was performed using an established interferon-responsive gene set,^9^ implemented in the gene set variation analysis package for R.^34^

### Role of the funding sources and study equipment providers

The funders and sponsor did not participate in the trial design, data accrual, analysis or manuscript preparation. The equipment provider (VitalTech) only participated in patient onboarding and in HIPAA-compliant data submission of deidentified primary data. Access to the raw data was available to the statisticians and study investigators. The corresponding author had full access to all data and the final responsibility for manuscript submission.

### Patient and public involvement

Patients or the public were not directly involved in this study, including but not limited to the trial design, patient recruitment, conduct of the trial, analysis of data and manuscript preparation.

### Data access

Cumulative longitudinal symptom data are available on reasonable request. The gene list for assessing type-I interferon response is provided in online supplemental table S5.

## Results

Between January 2021 and April 2021, 56 patients were enrolled (figure 1B), and 55 participants were included in the ITT analysis (placebo n=28; famotidine n=27). Forty-six (46) patients remained in the PP analysis (placebo n=27; famotidine n=19), and 35 patients had a sufficient number of high-quality RNA and plasma samples for the experimental medicine/exploratory analysis (placebo n=20; famotidine n=15). Overall, the missing data fraction over time was 9.9% in the ITT analysis (placebo 7.3%; famotidine 12.8%) and 5.6% in the PP analysis.

Table 1 summarises the comparison of patients’ baseline characteristics between the famotidine and placebo ITT groups. Female patients were almost two times as likely to enrol in the study as men. The study population was highly diverse, with matched enrollment of Black/African American (33%), mixed race (22%) and Hispanic (26%) patients. Patient level median baseline total symptom scores (median±IQR: famotidine: 18±13; placebo: 18±10) and average symptom durations prior to randomisation (median±IQR: famotidine: 4±2; placebo: 4±3) were closely matched also. Most patients had physiological measurements in the normal range, including average oxygen saturation at 99%. The characteristics for the PP and experimental medicine groups (online supplemental table S1A,B) were very similar to those of the ITT group.

**Table 1:** Patient and baseline characteristics from the intention-to-treat analysis

Variable	Level	Total (n=55)	Placebo (n=28)	Famotidine (n=27)	P value*
*Patients’ characteristics*					
Age (year)		35.0±20.0	31.5±13.0	35.0±18.0	0.162
Gender	Female	35 (63.6%)	18 (64.3%)	17 (63.0%)	0.919
	Male	20 (36.4%)	10 (35.7%)	10 (37.0%)	
Race	American Indian or Alaskan Native	1 (1.8%)	0 (0.0%)	1 (3.7%)	0.862
	Black or African American	18 (32.7%)	10 (35.7%)	8 (29.6%)	
	More than one race	12 (21.8%)	7 (25.0%)	5 (18.5%)	
	Unknown/not reported	2 (3.6%)	1 (3.6%)	1 (3.7%)	
	White	22 (40.0%)	10 (35.7%)	12 (44.4%)	
Ethnicity	Hispanic or Latino	14 (25.5%)	5 (17.9%)	9 (33.3%)	0.386
	Not Hispanic or Latino	22 (40.0%)	13 (46.4%)	9 (33.3%)	
	Unknown/not reported	19 (34.5%)	10 (35.7%)	9 (33.3%)	
*COVID-19 symptom score at baseline†*					
Total symptom score		18.0±11.0	18.0±13.0	18.0±10.0	0.985
*History of present illness*					
Symptomatic days prior to randomisation		4.0±3.0	4.0±2.0	4.0±3.0	0.363
*Vital signs at baseline‡*					
BMI (kg/m^2^)		27.33±7.78	25.43±8.28	28.93±6.69	0.149
Temperature (°F)		98.30±0.80	98.30±0.80	98.50±0.77	0.253
Heart rate (bpm)		88.0±19.0	87.0±23.0	89.0±16.5	0.385
SpO_2_ (%)		99.0±2.0	99.0±3.0	99.0±1.5	0.992
FEV_1_/FVC		0.88±0.23	0.88±0.19	0.88±0.25	0.614

For the continuous variable, median±IQR was reported.

*For categorical variables, p values were based on χ^2^ test with exact p value from Monte Carlo simulation; for the continuous variable, the p value was based on Wilcoxon rank sum test.

†Reason of missing data: IRB013 in placebo group, IRB041 and IRB052 in famotidine group withdrew early without baseline symptom scores.

‡Reason of missing data: IRB013 in placebo group, IRB041, IRB044, IRB052 in famotidine group withdrew early/were lost to follow-up without baseline vitals completed.

BMI, body mass index.

A total of 1358 electronic symptom surveys were submitted by the 55 patients in the ITT group (n=1215 for the PP group). Symptom frequency (counting symptoms with a score of at least 1) at baseline was equally distributed between the study arms (online supplemental figure S1C,D). Lack of energy, muscle pain, cough, runny nose and shortness of breath were most commonly reported by patients.

**Figure 2:**
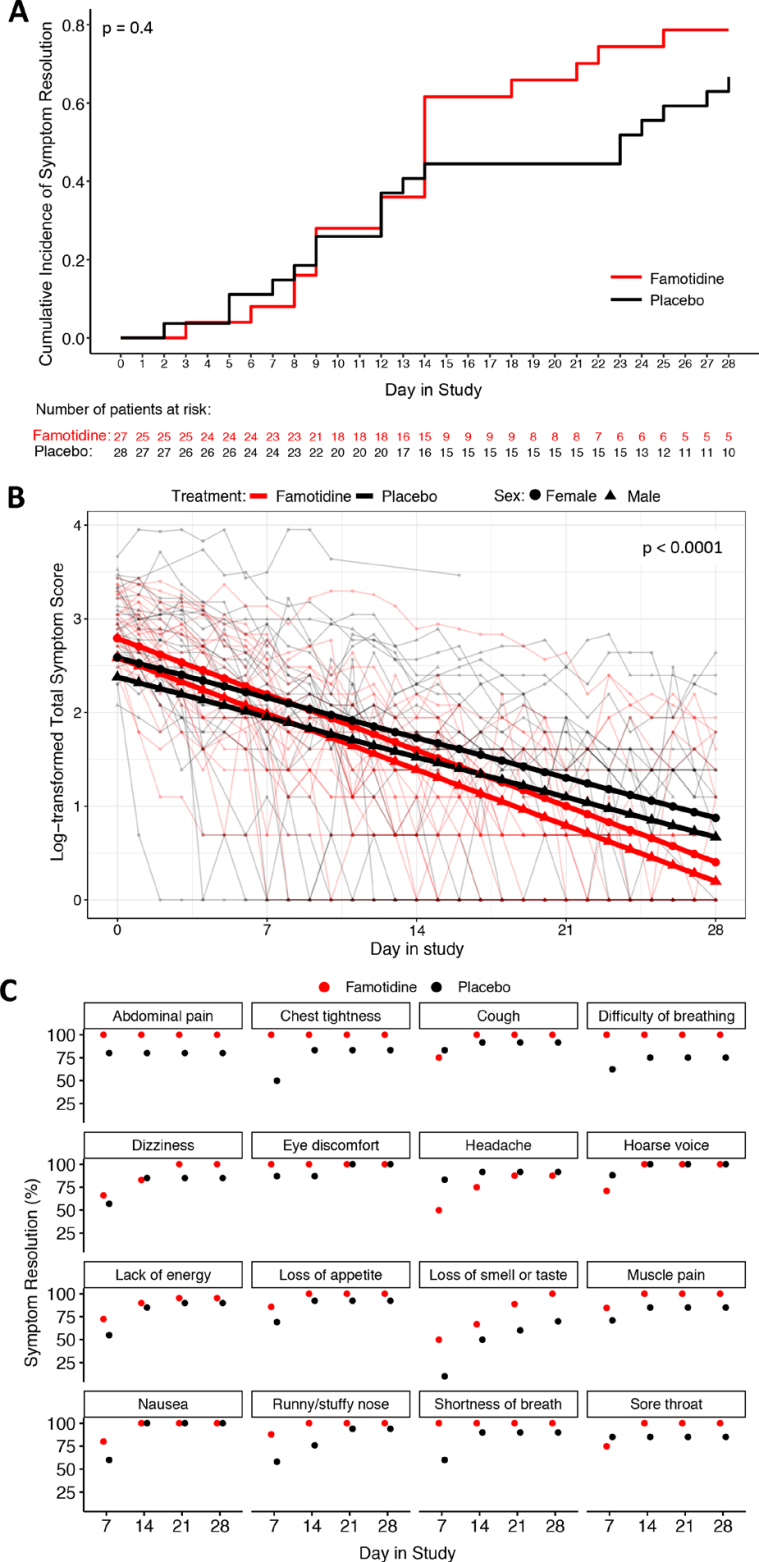
Intention-to-treat symptom resolution analyses. (A) The cumulative incidence of total symptom resolution for both study arms as defined in the primary trial endpoint is plotted. The famotidine and placebo arms were compared using stratified log-rank test. (B) The logarithmically transformed patient-level total symptom score (thin lines) and their estimated means based on linear mixed effect model are shown for each study arm. The p value for the interaction term of group and day in study is displayed. (C) The estimated cumulative incidence of symptom resolution for each individual symptom at days 7, 14, 21 and 28 is displayed for each study arm. The results for diarrhoea are not included because neither arm had symptomatic patients at the displayed timepoints. All timepoints with no remaining symptomatic patient are displayed as 100% symptom resolution.

The time to symptom resolution by study day 28 (primary endpoint) was not significantly different between patients in the famotidine and placebo arm in either the ITT (p value=0.4; figure 2A) or PP (p value=0.3; online supplemental figure S2A) analysis, although from day 14 onwards approximately two times as many patients remained symptomatic in the placebo group.

Comparison of the linear change rate in total symptom score, using the data from all 1358 longitudinal symptom scores submitted by the patients, revealed highly significantly different changing patterns between the two arms which was in favour of the famotidine arm. The estimated changing rates in ln(score+1) were −0.085 (95% CI: −0.099 to −0.071) in the famotidine group, and −0.061 (95% CI: −0.067 to −0.055) in the placebo group (p<0.0001; figure 2B). The estimated time to 50% symptom resolution was 8.2 days (95% CI: 7 to 9.8 days) for the famotidine and 11.4 days (95% CI: 10.3 to 12.6 days) for the placebo groups. The PP analyses delivered very similar statistically significant results in favour of the famotidine arm (online supplemental figure S2B). Importantly, further exploratory subgroup analysis showed that both sexes, and all race (p=0.6) and ethnicity (p=0.6) subgroups had a similar trend difference between the two study arms, however, the numbers of patients for this analysis were small. In the analysis for sex, the day in (study)*(treatment)*(sex) interaction term showed borderline greater benefit for male patients (p=0.07).

The impact on earlier symptom resolution of famotidine was examined on individual symptom level as secondary endpoint. The symptom diarrhoea was excluded, because it had low baseline prevalence and all patients were asymptomatic before day 7. Overall, patients on famotidine reported earlier symptom resolution for 14 out of 16 symptoms (87.5%), and those on placebo for 2 out of 16 symptoms (12.5%) (figure 2C, online supplemental table S2A). The PP analysis returned similar results (online supplemental figure S2C, table S2B). Among others, famotidine was associated with earlier resolution of lack of energy, loss of smell or taste, difficult of breathing, chest tightness and muscle pain while patients given the placebo experienced earlier resolution of headache, a known side effect of famotidine.

We next assessed the safety of famotidine. No severe adverse effects were observed. Five possibly medication-related adverse events were experienced during the 28-day study period, one each by five patients. Two patients, one from each arm, had mild elevations of serum markers of liver function. One patient in the famotidine arm had nausea and vomiting for 1 day, which settled after a 24-hour medication break and did not reoccur on restarting medication. In the placebo arm, one patient developed mild thrombocytopenia, and another patient had non-itching hives for 8 days. All of these events resolved spontaneously and were managed with interruption of study medication. We observed neutropenia (neutrophil count <1500/µL) in 11 patients. In the famotidine arm, all three patients with neutropenia on day 1 had normal neutrophil counts on day 7; in the placebo arm, 3 out of 8 patients with neutropenia on day 1 remained neutropenic at later timepoints.

With regard to clinical outcomes and their surrogates, no deaths, no hospitalisations and, therefore, no admissions to intensive care units occurred in either study arm. One emergency department assessment for symptomatic hypoxia during the study occurred in the placebo arm. Most laboratory measurements were in the normal range. No changes were detected for CRP (online supplemental table S3). However, in the ITT analysis, ferritin was significantly elevated on day 7 compared with day 1 in the placebo arm (+21%; p=0.008), but less so and not statistically significantly in the famotidine arm (+13%; p=0.09) (table 2). Both arms showed a statistically significant reduction in ferritin on day 28 compared with day 1 (33%; p<0.001 for both arms). The effect sizes were similar in the PP analysis (online supplemental table S4).

**Table 2:** Estimated mean and relative change in ferritin levels from the intention-to-treat analysis

Treatment	Visit	Estimated mean (95% CI)	Time	Relative change (95% CI)	P value*
Famotidine	Day 1	152.4 (111.0 to 209.4)			
	Day 7	171.5 (124.3 to 236.8)	Day 7 vs Day 1	0.13 (−0.02 to 0.29)	0.088
	Day 14	142.5 (102.8 to 197.5)	Day 14 vs Day 1	−0.07 (−0.19 to 0.08)	0.361
	Day 28	103.2 (74.7 to 142.4)	Day 28 vs Day 1	−0.32 (−0.41 to −0.23)	<0.0001
Placebo	Day 1	141.6 (102.9 to 194.9)			
	Day 7	170.6 (123.8 to 235.2)	Day 7 vs Day 1	0.21 (0.05 to 0.38)	0.008
	Day 14	129.7 (93.7 to 179.4)	Day 14 vs Day 1	−0.08 (−0.21 to 0.06)	0.226
	Day 28	95.5 (68.9 to 132.5)	Day 28 vs Day 1	−0.33 (−0.42 to −0.22)	<0.0001

*P values were based on t-test from a linear mixed model.

**Figure 3:**
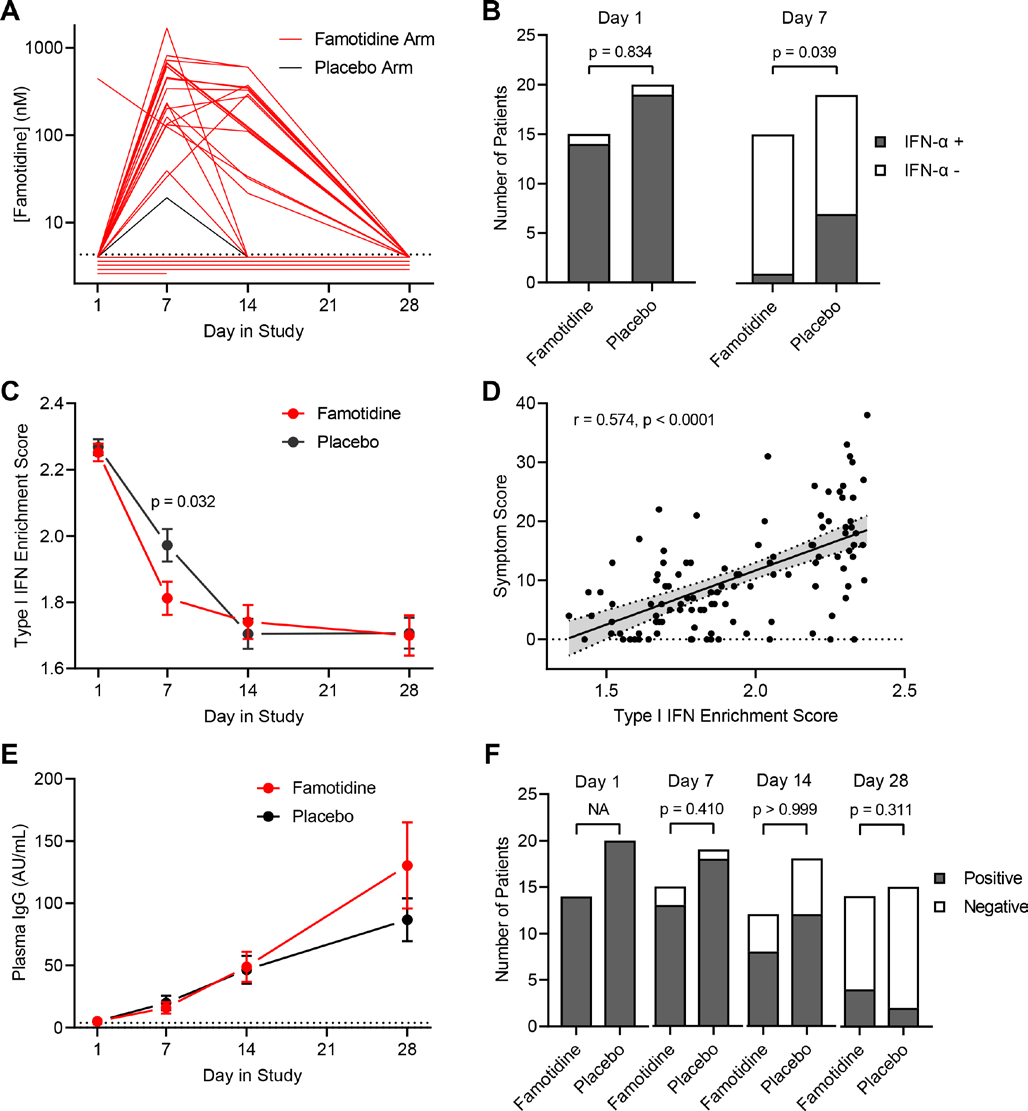
Effect of famotidine on inflammation and immunity. (A) The longitudinal plasma famotidine levels are displayed for patients enrolled in the famotidine arm and for the patient from the placebo arm with detectable plasma famotidine. (B) Participant numbers with detectable plasma interferon α levels in each arm at day 1 and day 7 of the trial are shown. Statistical comparison by χ^2^ test. (C) Enrichment scores for type-I interferon response genes expressed in peripheral blood mononuclear cells (PBMCs) at days 1, 7, 14 and 28 are shown. (D) The correlation of symptom score and enrichment scores for type-I interferon is assessed. Statistical comparison by Spearman rank analysis. (E) The levels (mean and SE of the mean) of class g immunoglobulins reactive to the SARS-CoV-2 core protein are plotted for days 1, 7, 14 and 28 for each study arm. (F) The number of study participants with RT-PCR-detectable viral RNA extracted from nasal swabs on days 1, 7, 14 and 28 are shown for each trial arm. IFN: interferon, IgG: immunoglobulin type G.

**Figure 4:**
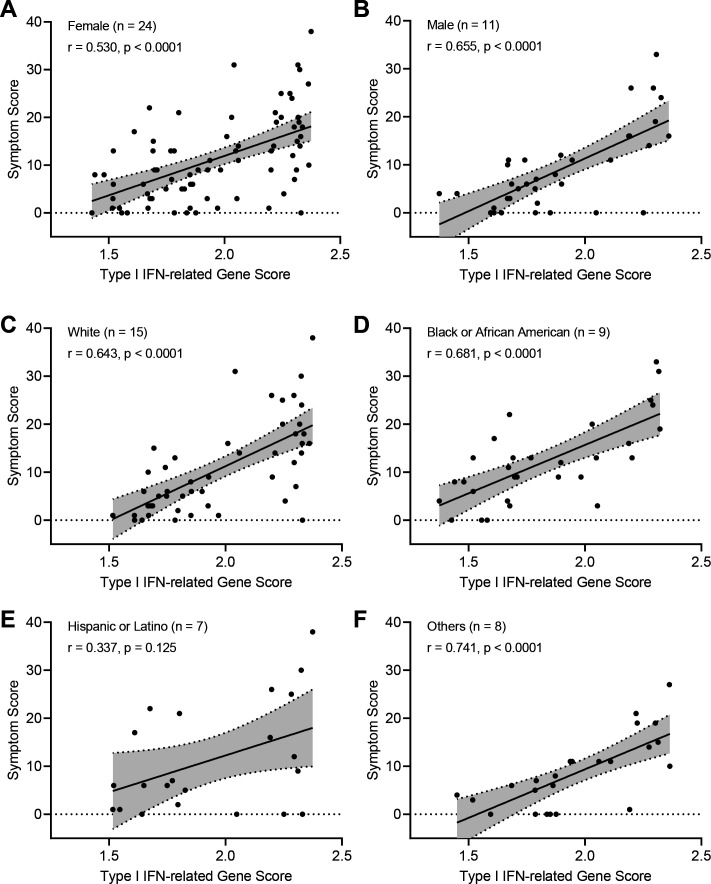
Analysis of symptom score and type-I interferon signal correlation at patient category level. (A–F) The correlation of symptom score and enrichment scores for type-I interferon was assessed for the indicated group of patients. Patient counts for each group are displayed. Patients were able to self-assign to more than one race/ethnicity group, and the total patient count for panel C–F is therefore larger than 35, the total number of patients in the experimental medicine section of the trial. Statistical comparison by Spearman rank analysis. IFN: interferon.

To examine the biological effect of famotidine in the experimental medicine group of the study, we identified patients for inclusion by determining plasma famotidine levels during the treatment period using mass spectrometry and RNA availability from PBMCs (figures 1B and 3A). Five patients in the famotidine arm did not show measurable plasma famotidine, indicating likely non-adherence to the medication; while one patient in the placebo arm showed smaller but evident plasma famotidine level on day 7, possibly due to over-the-counter use of famotidine. Plasma famotidine levels for patients in the famotidine arm were in the range of 0.02–1.69 µM (IQR=0.13–0.57 µM). These levels agree with the pharmacokinetics of oral intake of 80 mg famotidine three times a day and a wide sampling interval range due to the variety of mobile phlebotomy timing in relation to medication intake and famotidine’s elimination half-life of 4 hours.^35^ On study day 1, most patients in both study arms had detectable interferon alpha plasma levels, but significantly fewer patients on famotidine had measurable plasma interferon alpha on day 7 (p=0.039; figure 3B). To orthogonally validate this finding, we performed RNA sequencing on patient PBMCs and computed a type-I interferon gene score comprising 29 genes (online supplemental table S5), which was significantly reduced on day 7 in patients taking famotidine (p=0.032; figure 3C). The transcript levels of 2’−5’ oligoadenylate synthetases (OAS1–3), important second messengers of type-I interferons, were also significantly reduced on day 7 in patients taking famotidine (online supplemental figure S3). The type-I interferon score was correlated with the total symptom score, suggesting that patient-reported symptom severity is linked to sustained interferon-mediated inflammation (figure 3D) an observation that was made for female, male, white, black, Hispanic and other groups of patients (figure 4). To investigate the effect of famotidine on adaptive immunity, we quantified longitudinal plasma immunoglobulin type G (IgG) levels to SARS-CoV-2 nucleocapsid core protein (figure 3E) and viral clearance by RT-PCR detection of viral RNA from deep nasal swabs (figure 3F), finding no significant differences between both study arms.

## Discussion

This randomised, double-blind and placebo-controlled phase 2 clinical trial examined the effect of oral famotidine (80 mg three times a day) taken for 14 days by a diverse patient population with mild to moderate COVID-19. In concordance with pre-clinical mechanistic work,^9^ we found that famotidine leads to earlier resolution of type-I interferon elevation, without reduced anti-viral immunity. Famotidine improved resolution of 14 out of 16 assessed symptoms and led to a statistically significant increased rate of symptom recovery.

Our study has limitations. Although the chosen primary endpoint of symptom resolution was not met at statistical threshold, this analysis showed that twice as many patients stayed symptomatic on the placebo arm from day 14 onwards. In addition, most patients of our study, by physiological and standard laboratory measures, had mild disease only. Follow-up studies should perhaps focus on patients with moderate disease severity or patients with symptomatic deterioration. At the time of our trial, patients over the age of 65 or over 55 years with comorbidities received antibody therapy, which in turn led to exclusion from this study. Also, our patients had not received vaccinations to SARS-CoV-2 prior to or during the study and were not infected by the variants of SARS-CoV-2 that currently cause most global cases. Nevertheless, our results are likely to be less affected by the emergence of viral variants and more relevant to patients with breakthrough infections than findings from studies related to vaccinations or anti-viral therapies^17 36^ because the evidence strongly suggests that famotidine targets the host and not the virus.^9 37^

The power of a dataset in clinical trials, an essential consideration in the study design, is not simply based on the number of enrolled patients. Studies that use rare events in patients as endpoints, for example hospitalisation or death in patients with mild to moderately severe COVID-19, require large patient numbers to generate a small number of endpoint-related events.^24^ We acquired and used intensive longitudinal data^38^ from every patient. Thus, 1358 data points from 55 patients were used for the changing rate of symptom score modelling to examine differences in two arms. Operationally, using more data from fewer patients means that more institutions can independently participate in therapeutic hypothesis testing. Our work demonstrates that a fully remote trial that includes acquisition of biological samples can be delivered at reasonable cost (estimated total cost <1.5M US$) and in a short timeframe. We combined pharmacological confirmation of treatment concordance with biological readouts of the drug-induced resolution of inflammation and patient-reported symptoms in a fully remote, double-blind, placebo-controlled trial. This may be a template for future early phase clinical trials for COVID-19 and perhaps other diseases. In fact, we find a generalised strong correlation of symptom score and inflammatory score, that indicates the option to conducted informative COVID-19 treatment trials using patient-reported longitudinal outcome measures and linear mixed models to assess treatment effect. This is a relevant consideration for the inclusion of healthcare systems with less resources. More treatments could be tested by globally distributed research groups if our study paradigm was used. Studies could also expand to important groups with fewer patients, such as pregnant and paediatric patients, who are often poorly covered by clinical trials, including for COVID-19. For example, evidence-based treatment options for pregnant women with COVID-19 and for the COVID-19-induced multisystem inflammatory syndrome in children are needed, and famotidine is safe during pregnancy and in children though doses will need to be optimised.^35^

Our study has additional strengths. Famotidine is a safe, low-cost and widely available medication with excellent tolerability and minimal known drug–drug interactions^35^ that has been taken by millions of patients worldwide. These characteristics are relevant because the number-needed-to-treat to prevent one serious event is high in non-hospitalised patients. Therefore, treatments should have minimal risks, side effects and costs. Our findings should generalise well, given the balanced group of patients from diverse backgrounds, including African American and Hispanic patients, who are at higher risk of poor outcome from COVID-19.^13^ We also note that patients in the placebo arm were at higher risk of remaining symptomatic, indicating that famotidine should perhaps be investigated for prevention of long COVID-19. In fact, sustained inflammation may be detrimental in viral diseases^39^ other than COVID-19, and the biological effect of H2R blockade may well be transferrable.

In accordance with mechanistic laboratory studies,^9^ the blockade of H2R causes detectable early recovery of elevated interferon alpha levels in the plasma, presumably by modulating inflammation in organ tissues. Interferon alpha has been linked to ferritin upregulation in non-COVID-19-related clinical studies.^40 41^ The larger increase of ferritin on day 7 in the placebo arm of this study can therefore be explained by a mechanistic sequence. Ferritin has been suggested as a biomarker for COVID-19^42^ and could be used in future famotidine trials.

Important information in this study was gathered by determining plasma famotidine levels. Measured treatment concordance was 76% in keeping with other outpatient trials.^43^ One patient on the placebo arm had detectable famotidine levels, presumably due to inadvertent intake of over-the-counter famotidine. Due to study design, that is, use of plasma famotidine levels for PP population identification, the drop-out rate for the placebo arm in the PP group was smaller than for the famotidine arm. The plasma famotidine levels were mostly higher than the reported IC50 levels for famotidine-mediated H2R blockade (0.039 µM).^44^ Therefore, the famotidine dose of 80 mg orally administered three times a day likely achieves sustained H2R blockade. In contrast, high-dose administration of other H2R receptor antagonists, such as cimetidine, may not lead to sufficient H2R blockade,^9^ perhaps explaining why retrospective cohort studies have not identified a class effect.

The type-I interferon response has a dual role in COVID-19, and the chronicity of type-I interferon release needs to be carefully considered.^23 45^ Initial elevations are essential for effective immunity,^46 47^ and patients with inborn errors of type-I interferon immunity or anti-interferon alpha antibodies are at higher risk of severe COVID-19.^48 49^ These results suggest a conceptual risk of high-dose famotidine treatment if taken prophylactically, that is, prior to the initiation of anti-viral immunity. Famotidine for treating reflux is taken at low doses of 10–40 mg a day, which is probably insufficient to suppress anti-viral type-I interferon responses. This may explain why famotidine has been found to effectively reduce the HR of death, intubation or critical complications in some retrospective cohort studies,^25 26^ but not in others.^27^ On the other hand, sustained interferon alpha elevation results in damage to non-infected tissue and worse clinical outcome.^50^ In mouse models of SARS-CoV and MERS-CoV infection, a delayed interferon response is associated with more severe symptoms and poorer outcome.^51 52^ Similarly, in patients, delayed administration of inhaled interferon alpha has been linked to worsened clinical outcome.^53^ Altogether, these findings suggest a model in which high-dose famotidine treatment—given after symptom onset and outside the time window when interferon responses are essential for controlling viremia—can improve outcome without increasing the risk of hospitalisation or death. Future studies should include initiation of famotidine administration to patients with delayed onset of symptomatic disease, that is, those with positive PCR tests more than 7 days prior to symptom development.

Famotidine is likely to remain a drug used for COVID-19, either prescribed by physicians or self-administered by patients. Our finding may support this use of famotidine, given that we show that famotidine is well tolerated and that it accelerates the resolution of symptoms and inflammation without compromising immunity. We acknowledge that additional research studies and clinical trials remain a priority.

## Data Availability

Data are available upon reasonable request. The gene list for assessing type-I interferon response is provided in Table S5.
