# Supplementary material for: Oral famotidine versus placebo in non-hospitalised patients with COVID-19: a randomised, double-blind, data-intense, phase 2 clinical trial
Source: Gut. 2022 Feb 10;71(5):879–88. doi: 10.1136/gutjnl-2022-326952 (PMC8844971; doi:10.1136/gutjnl-2022-326952)
Supplement: Supplementary data [file gutjnl-2022-326952supp001.pdf]

SUPPLEMENTAL MATERIAL

Oral Famotidine vs. Placebo in Diverse Non-Hospitalized Patients with COVID-19: A Randomized, Double-Blinded Phase 2 Clinical Trial

Brennan *et al.*

Extended Methods

Study design. The data were gathered via FDA-approved Bluetooth-compatible devices: Jumper Pulse Oximeter, Jumper thermometer, MIR Spirobank II spirometer, and Taiza weight scale. Electronic devices and study medication were sent to the patient at their place of residence by overnight delivery after consent (day 0). Blood samples were processed and analyzed at a central laboratory. Research samples were processed, snap frozen, and stored at -80°C until use.

Randomization and masking. Unblinding of the investigators and determining plasma famotidine levels occurred only after the final report of the clinical endpoints had been reviewed and accepted by the DSMB.

Treatment. Capsules in sterile, sealed bottles labeled with the blinded code for the study arm were shipped to study participants together with the remote monitoring devices. Self-reported concordance was documented on study days 7 and 14.

Statistical analysis

Cumulative incidence of symptom resolution at day 28: The analysis did not consider any competing risk event, as no death event occurred.

Plasma Interferon-α (IFN- α) level

Brennan CM, *et al. Gut* 2022; 71:879–888. doi: 10.1136/gutjnl-2022-326952

Plasma IFN-α concentrations were determined using VeriKine-HS Human Interferon Alpha All Subtype ELISA kit (Cat. No. 41115-1, PBL Assay Science, New Jersy, USA) and read on a SpectraMax i3x microplate reader (Molecular Devices, California, USA). Samples were diluted if optical reading exceeded the upper linear range.

Data access

The transcriptional data will be made available at the point of publication of the peer-reviewed manuscript.

Brennan CM, *et al. Gut* 2022; 71:879–888. doi: 10.1136/gutjnl-2022-326952

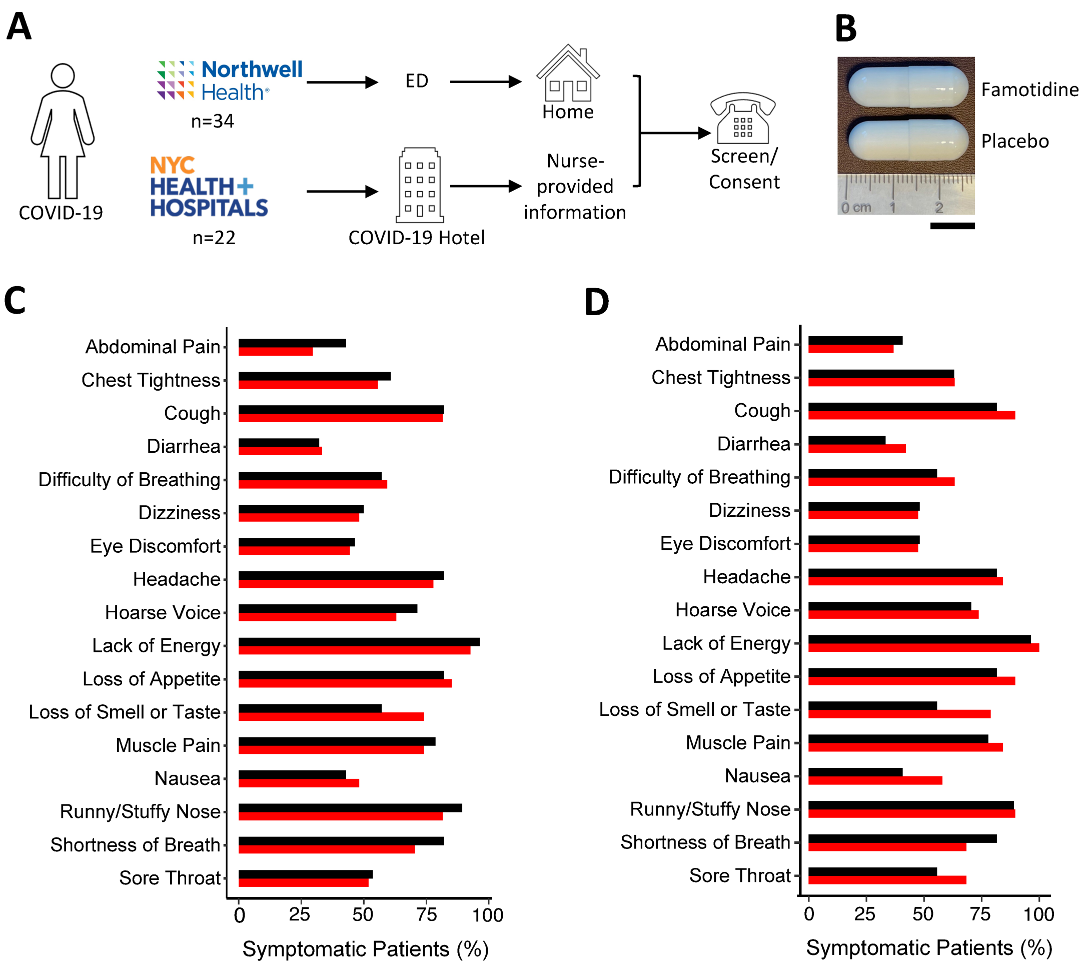

Figure S1. Extended trial overview and baseline symptom scores. (**A**) The candidate patient identification schematic is shown. (**B**) A photograph of example capsules filled with famotidine or placebo is displayed. Scale bar: 10mm. (**C-D**) The symptom frequencies at baseline for all 17 assessed individual symptoms are shown for each study arm for the (**C**) ITT and (**D**) PP group. Black bars: placebo, red bars: famotidine

ED = emergency department.

Brennan CM, *et al. Gut* 2022; 71:879–888. doi: 10.1136/gutjnl-2022-326952

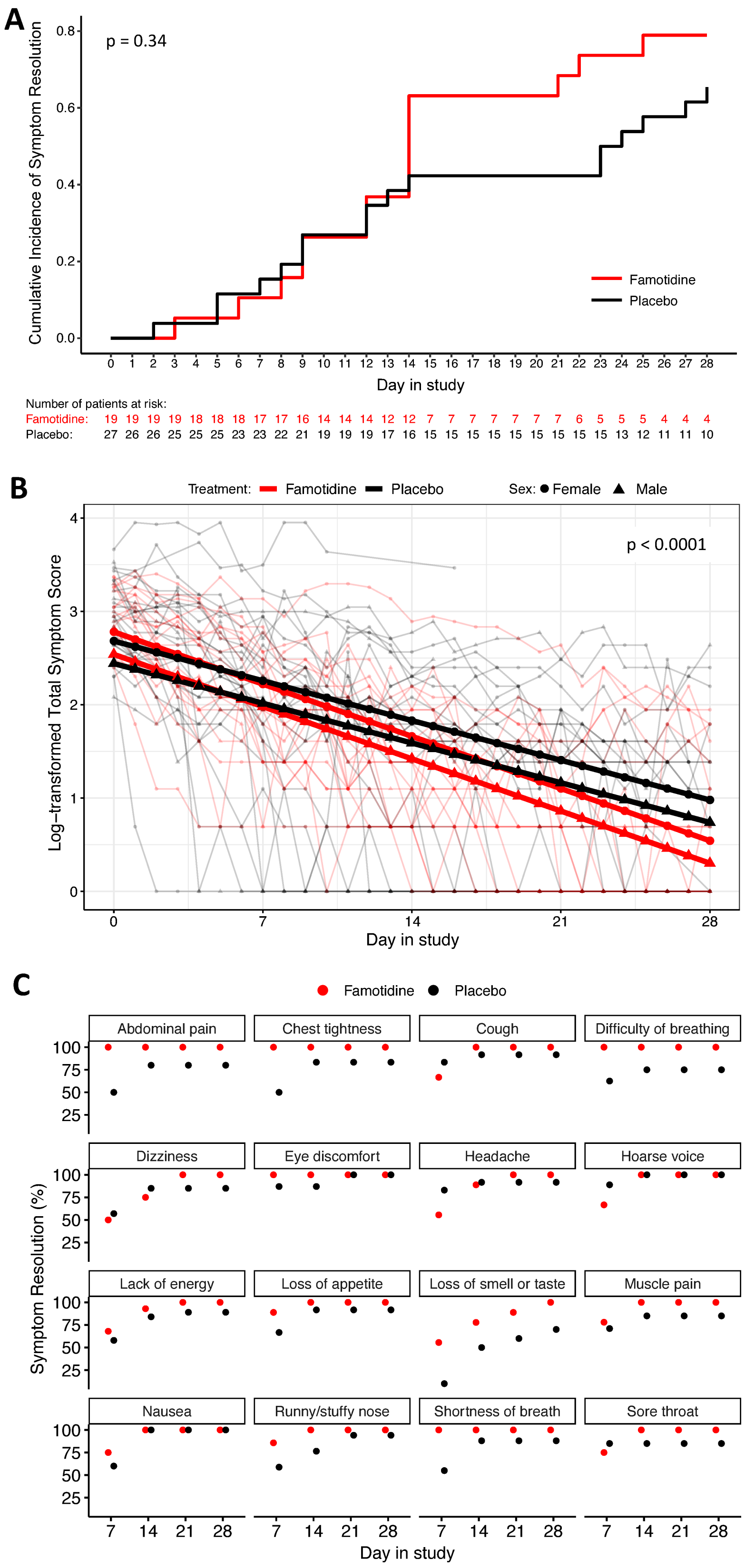
A
p = 0.34
Cumulative Incidence of Symptom Resolution
Famotidine
Placebo
Day in study
Number of patients at risk:
Famotidine: 19 19 19 19 18 18 18 17 17 16 14 14 14 12 12 7 7 7 7 7 7 7 6 5 5 5 4 4 4
Placebo: 27 26 26 25 25 25 23 23 22 21 19 19 19 17 16 15 15 15 15 15 15 15 15 15 13 12 11 11 10
B
Treatment: Famotidine Placebo
Sex: Female Male
p < 0.0001
Log−transformed Total Symptom Score
Day in study
C
Famotidine Placebo
Abdominal pain
Chest tightness
Cough
Difficulty of breathing
Dizziness
Eye discomfort
Headache
Hoarse voice
Lack of energy
Loss of appetite
Loss of smell or taste
Muscle pain
Nausea
Runny/stuffy nose
Shortness of breath
Sore throat
Symptom Resolution (%)
Day in study

4

47

Figure S2. Per-protocol symptom resolution analyses. (**A**) The cumulative incidence of total symptom resolution for both study arms as defined in the primary trial endpoint is plotted. The famotidine and placebo arms were compared using stratified log-rank test. (**B**) The logarithmically transformed patient-level total symptom score (thin lines) and their estimated means based on linear mixed effect model are shown for each study arm. The p-value for the interaction term of group and day in study is displayed. (**C**) The estimated cumulative incidence of symptom resolution for each individual symptom at day 7, 14, 21, and 28 are displayed for each study arm. The results for diarrhea are not included because neither arm had symptomatic patients at the displayed timepoints. All timepoints with no remaining symptomatic patient are displayed as 100% symptom resolution.

Brennan CM, *et al. Gut* 2022; 71:879–888. doi: 10.1136/gutjnl-2022-326952

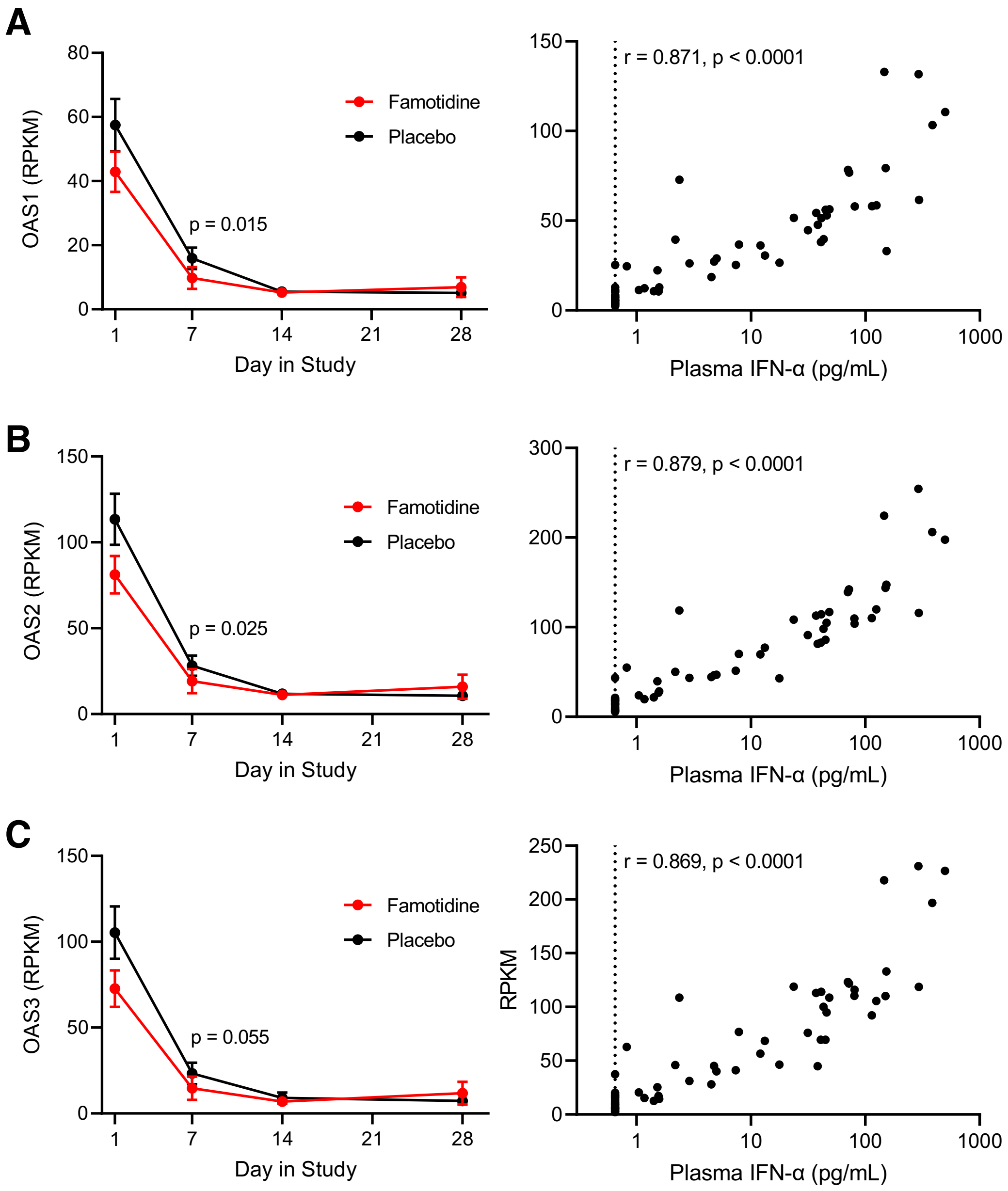

Figure S3. Analysis of 2´-5´oligoadenylate synthetase (OAS) gene expression in PBMCs. (A-C) The expression levels of OAS genes (*OAS1*, *OAS2* and *OAS3*) in PBMCs at day 1, 7, 14, 28 (left panel) and the correlation of OAS gene expression and plasma IFN-α levels (right panel) are shown. Dashed vertical lines indicate the lower limit of detection (LLD), and all IFN-α measuremnts falling below were treated as LLD. Gene expression levels between famotidine and placebo arms were compared by Wilcoxon rank sum test. P-values for day 7 are displayed. For all other comparisons at day 1, 14 and 28, p values were greater than 0.2. Statistical comparison of correlation was by Spearman rank analysis.

Table S1A. Patient and baseline characteristics from the per-protocol analysis.

Variable	Level	Total (N=46)	Placebo (N=27)	Famotidine (N=19)	P-value*
**Patients' characteristics**					
Age (year)		35.0±19.0	31.0±13.0	39.0±18.0	0.113
Gender	Female	30 (65.2%)	17 (63.0%)	13 (68.4%)	0.702
	Male	16 (34.8%)	10 (37.0%)	6 (31.6%)	
Race	Black or African American	15 (32.6%)	10 (37.0%)	5 (26.3%)	0.655
	More than one race	10 (21.7%)	7 (25.9%)	3 (15.8%)	
	Unknown/not reported	2 (4.4%)	1 (3.7%)	1 (5.3%)	
	White	19 (41.3%)	9 (33.3%)	10 (52.6%)	
Ethnicity	Hispanic or Latino	11 (23.9%)	5 (18.5%)	6 (31.6%)	0.455
	Not Hispanic or Latino	19 (41.3%)	13 (48.2%)	6 (31.6%)	
	Unknown/not reported	16 (34.8%)	9 (33.3%)	7 (36.8%)	
**COVID-19 Symptom Score at Baseline****					
Total Symptom Score		18.0±11.0	18.0±13.0	18.0±11.0	0.927
**History of Present Illness**					
Symptomatic days prior to randomization		4.0±3.0	4.0±2.0	4.0±3.0	0.339
**Vital Signs at Baseline****					
BMI (kg/m^2)		26.75±7.33	24.94±8.28	27.48±6.79	0.158
Temperature (°F)		98.40±0.80	98.30±0.80	98.60±0.80	0.269
Heart Rate (bpm)		87.0±16.0	86.5±21.0	88.0±16.0	0.370
SpO2(%)		99.0±2.0	99.0±3.0	99.0±2.0	0.812
FEV1/FVC		0.89±0.21	0.88±0.18	0.89±0.29	0.465

*: For categorical variables, p-values were based on Chi-squared test with exact p-value from Monte Carlo simulation; for the continuous variable, the p-value was based on Wilcoxon rank sum test.
**: Reason of missing data: IRB013 in the placebo group withdrew early without baseline symptom scores and vitals completed.
Note: For the continuous variable, median+/-IQR was reported.

Table S1B. Patient and baseline characteristics from the experimental medicine analysis.

Variable	Level	Total (N=35)	Placebo (N=20)	Famotidine (N=15)	P-value*
**Patients' characteristics**					
Age (year)		36.0±17.0	33.5±14.5	41.0±17.0	0.182
Gender	Female	24 (68.6%)	15 (75.0%)	9 (60.0%)	0.344
	Male	11 (31.4%)	5 (25.0%)	6 (40.0%)	
Race	Black or African American	9 (25.7%)	6 (30.0%)	3 (20.0%)	0.369
	More than one race	9 (25.7%)	7 (35.0%)	2 (13.3%)	
	Unknown/not reported	2 (5.7%)	1 (5.0%)	1 (6.7%)	
	White	15 (42.9%)	6 (30.0%)	9 (60.0%)	
Ethnicity	Hispanic or Latino	7 (20.0%)	3 (15.0%)	4 (26.7%)	0.492
	Not Hispanic or Latino	14 (40.0%)	10 (50.0%)	4 (26.7%)	
	Unknown/not reported	14 (40.0%)	7 (35.0%)	7 (46.7%)	
**COVID-19 Symptom Score at Baseline**					
Total Symptom Score		19.0±11.0	18.5±13.5	19.0±9.0	0.789
**History of Present Illness**					
Symptomatic days prior to randomization		4.00±2.0	3.00±1.5	4.00±3.0	0.198
**Vital Signs at Baseline**					
BMI (kg/m^2)		26.73±12.79	25.99±13.17	27.27±6.79	0.395
Temperature (°F)		98.40±0.80	98.25±0.75	98.60±0.80	0.404
Heart Rate (bpm)		87.0±15.0	85.0±23.5	88.0±11.0	0.582
SpO2(%)		99.0±2.0	99.0±2.0	99.0±2.0	0.704
FEV1/FVC		0.89±0.21	0.90±0.16	0.89±0.29	0.545

*: For categorical variables, p-values were based on Chi-squared test with exact p-value from Monte Carlo simulation; for the continuous variable, the p-value was based on Wilcoxon rank sum test.
Note: For the continuous variable, median+/-IQR was reported.

Table S2A. Estimated cumulative incidences (CIN) of individual symptom resolution and corresponding 95% confidence intervals for symptom resolution at day 7, 14, 21, 28 from the intention-to-treat analysis.

Treatment	Day 7	Day 14	Day 21	Day 28
**Lack of energy**				
Famotidine	72.7% (47.8%, 87.2%)	90.9% (62.7%, 98.1%)	95.5% (55.6%, 99.6%)	95.5% (55.6%, 99.6%)
Placebo	55.0% (30.4%, 74.1%)	85.0% (56.9%, 95.4%)	90.0% (59.6%, 97.9%)	90.0% (59.6%, 97.9%)
**Shortness of breath**				
Famotidine	NSP	NSP	NSP	NSP
Placebo	60.0% (22.2%, 84.1%)	90.0% (25.4%, 99.2%)	90.0% (25.4%, 99.2%)	90.0% (25.4%, 99.2%)
**Cough**				
Famotidine	75.0% (23.4%, 94.5%)	NSP	NSP	NSP
Placebo	83.3% (40.8%, 96.4%)	91.7% (32.9%, 99.3%)	91.7% (32.9%, 99.3%)	91.7% (32.9%, 99.3%)
**Headache**				
Famotidine	50.0% (19.3%, 74.7%)	75.0% (37.3%, 91.9%)	87.5% (23.5%, 98.8%)	87.5% (23.5%, 98.8%)
Placebo	83.3% (40.8%, 96.4%)	91.7% (32.9%, 99.3%)	91.7% (32.9%, 99.3%)	91.7% (32.9%, 99.3%)
**Loss of smell or taste**				
Famotidine	50.0% (19.3%, 74.7%)	66.7% (30.7%, 87.0%)	88.9% (23.8%, 99.0%)	NSP
Placebo	10.0% (0.5%, 37.4%)	50.0% (16.3%, 76.7%)	60.0% (22.2%, 84.1%)	70.0% (28.2%, 90.4%)
**Loss of appetite**				
Famotidine	85.7% (46.8%, 96.9%)	NSP	NSP	NSP
Placebo	69.2% (34.3%, 88.1%)	92.3% (35.8%, 99.4%)	92.3% (35.8%, 99.4%)	92.3% (35.8%, 99.4%)
**Difficulty of breathing**				
Famotidine	NSP	NSP	NSP	NSP
Placebo	62.5% (19.0%, 87.5%)	75.0% (23.6%, 94.4%)	75.0% (23.6%, 94.4%)	75.0% (23.6%, 94.4%)
**Diarrhea**				
Famotidine	NSP	NSP	NSP	NSP
Placebo	NSP	NSP	NSP	NSP
**Sore throat**				
Famotidine	75.0% (1.9%, 97.9%)	NSP	NSP	NSP
Placebo	85.7% (13.0%, 98.8%)	85.7% (13.0%, 98.8%)	85.7% (13.0%, 98.8%)	85.7% (13.0%, 98.8%)
**Muscle pain**				
Famotidine	84.6% (44.1%, 96.7%)	NSP	NSP	NSP

Brennan CM, *et al. Gut* 2022; 71:879–888. doi: 10.1136/gutjnl-2022-326952

Treatment	Day 7	Day 14	Day 21	Day 28
Placebo	71.4% (38.0%, 89.0%)	85.7% (48.2%, 96.8%)	85.7% (48.2%, 96.8%)	85.7% (48.2%, 96.8%)
**Hoarse voice**				
Famotidine	71.4% (18.3%, 93.6%)	NSP	NSP	NSP
Placebo	89.0% (29.2%, 98.9%)	NSP	NSP	NSP
**Runny/stuffy nose**				
Famotidine	88.2% (56.3%, 97.3%)	NSP	NSP	NSP
Placebo	58.8% (31.2%, 78.5%)	76.5% (46.1%, 91.1%)	94.1% (47.1%, 99.5%)	94.1% (47.1%, 99.5%)
**Chest tightness**				
Famotidine	NSP	NSP	NSP	NSP
Placebo	50.0% (7.7%, 82.9%)	83.3% (8.6%, 98.7%)	83.3% (8.6%, 98.7%)	83.3% (8.6%, 98.7%)
**Abdominal pain**				
Famotidine	NSP	NSP	NSP	NSP
Placebo	80.0% (4.7%, 98.4%)	80.0% (4.7%, 98.4%)	80.0% (4.7%, 98.4%)	80.0% (4.7%, 98.4%)
**Nausea**				
Famotidine	80.0% (5.3%, 98.3%)	NSP	NSP	NSP
Placebo	60.0% (6.7%, 90.8%)	NSP	NSP	NSP
**Dizziness**				
Famotidine	66.7% (12.2%, 92.5%)	83.3% (8.6%, 98.7%)	NSP	NSP
Placebo	57.1% (13.2%, 85.7%)	85.7% (12.9%, 98.9%)	85.7% (12.9%, 98.9%)	85.7% (12.9%, 98.9%)
**Eye discomfort**				
Famotidine	NSP	NSP	NSP	NSP
Placebo	87.5% (24.6%, 98.7%)	87.5% (24.6%, 98.7%)	NSP	NSP

NSP: No symptomatic patient, corresponding to 100% cumulative symptom resolution.

Table S2B. Estimated cumulative incidences (CIN) of individual symptom resolution and corresponding 95% confidence intervals for symptom resolution at day 7, 14, 21, 28 from the per-protocol analysis.

Treatment	Day 7	Day 14	Day 21	Day 28
**Lack of energy**				
Famotidine	68.8% (38.7%, 86.2%)	93.8% (44.1%, 99.5%)	NSP	NSP
Placebo	57.9% (32.1%, 76.9%)	84.2% (55.0%, 95.2%)	89.5% (57.9%, 97.8%)	89.5% (57.9%, 97.8%)
**Shortness of breath**				
Famotidine	NSP	NSP	NSP	NSP
Placebo	55.6% (17.5%, 82.0%)	88.9% (21.5%, 99.1%)	88.9% (21.5%, 99.1%)	88.9% (21.5%, 99.1%)
**Cough**				
Famotidine	66.7% (12.3%, 92.5%)	NSP	NSP	NSP
Placebo	83.3% (40.8%, 96.4%)	91.7% (32.9%, 99.3%)	91.7% (32.9%, 99.3%)	91.7% (32.9%, 99.3%)
**Headache**				
Famotidine	55.6% (17.7%, 81.9%)	88.9% (32.9%, 98.8%)	NSP	NSP
Placebo	83.3% (40.8%, 96.4%)	91.7% (32.9%, 99.3%)	91.7% (32.9%, 99.3%)	91.7% (32.9%, 99.3%)
**Loss of smell or taste**				
Famotidine	55.6% (17.8%, 81.9%)	77.8% (28.3%, 95.1%)	88.9% (21.5%, 99.1%)	NSP
Placebo	10.0% (0.5%, 37.4%)	50.0% (16.3%, 76.7%)	60.0% (22.2%, 84.1%)	70.0% (28.2%, 90.4%)
**Loss of appetite**				
Famotidine	88.9% (21.5%, 99.1%)	NSP	NSP	NSP
Placebo	66.7% (30.7%, 87.0%)	91.7% (32.6%, 99.3%)	91.7% (32.6%, 99.3%)	91.7% (32.6%, 99.3%)
**Difficulty of breathing**				
Famotidine	NSP	NSP	NSP	NSP
Placebo	62.5% (19.0%, 87.5%)	75.0% (23.6%, 94.4%)	75.0% (23.6%, 94.4%)	75.0% (23.6%, 94.4%)
**Diarrhea**				
Famotidine	NSP	NSP	NSP	NSP
Placebo	NSP	NSP	NSP	NSP
**Sore throat**				
Famotidine	75.0% (1.9%, 97.9%)	NSP	NSP	NSP
Placebo	85.7% (13.0%, 98.8%)	85.7% (13.0%, 98.8%)	85.7% (13.0%, 98.8%)	85.7% (13.0%, 98.8%)
**Muscle pain**				
Famotidine	77.8% (30.2%, 94.9%)	NSP	NSP	NSP
Placebo	71.4% (38.0%, 89.0%)	85.7% (48.2%, 96.8%)	85.7% (48.2%, 96.8%)	85.7% (48.2%, 96.8%)

Treatment	Day 7	Day 14	Day 21	Day 28
**Hoarse voice**				
Famotidine	66.7% (13.7%, 92.1%)	NSP	NSP	NSP
Placebo	88.9% (29.2%, 98.9%)	NSP	NSP	NSP
**Runny/stuffy nose**				
Famotidine	85.7% (48.1%, 96.9%)	NSP	NSP	NSP
Placebo	58.8% (31.2%, 78.5%)	76.5% (46.1%, 91.1%)	94.1% (47.1%, 99.5%)	94.1% (47.1%, 99.5%)
**Chest tightness**				
Famotidine	NSP	NSP	NSP	NSP
Placebo	50.0% (7.7%, 82.9%)	83.3% (8.6%, 98.7%)	83.3% (8.6%, 98.7%)	83.3% (8.6%, 98.7%)
**Abdominal pain**				
Famotidine	NSP	NSP	NSP	NSP
Placebo	80.0% (4.7%, 98.4%)	80.0% (4.7%, 98.4%)	80.0% (4.7%, 98.4%)	80.0% (4.7%, 98.4%)
**Nausea**				
Famotidine	75.0% (1.9%, 97.9%)	NSP	NSP	NSP
Placebo	60.0% (6.7%, 90.8%)	NSP	NSP	NSP
**Dizziness**				
Famotidine	50.0% (2.3%, 88.1%)	75.0% (1.7%, 98.0%)	NSP	NSP
Placebo	57.1% (13.2%, 85.7%)	85.7% (12.9%, 98.9%)	85.7% (12.9%, 98.9%)	85.7% (12.9%, 98.9%)
**Eye discomfort**				
Famotidine	NSP	NSP	NSP	NSP
Placebo	87.5% (24.6%, 98.7%)	87.5% (24.6%, 98.7%)	NSP	NSP

NSP: No symptomatic patient, corresponding to 100% cumulative symptom resolution.

Table S3A Estimated mean and relative change in CRP levels from the intention-to-treat analysis.

Treatment	Visit	Estimated mean (95% CI)	Time	Relative change (95% CI)	P-value*
Famotidine	Day 1	0.919 (0.540, 1.565)			
	Day 7	0.954 (0.510, 1.784)	Day 7 vs Day 1	0.038 (-0.494, 1.131)	0.917
	Day 14	0.563 (0.308, 1.030)	Day 14 vs Day 1	-0.388 (-0.698, 0.242)	0.169
	Day 28	0.621 (0.329, 1.17)	Day 28 vs Day 1	-0.325 (-0.675, 0.402)	0.284
Placebo	Day 1	0.517 (0.302, 0.884)			
	Day 7	0.958 (0.474, 1.933)	Day 7 vs Day 1	0.853 (-0.164, 3.106)	0.125
	Day 14	0.261 (0.108, 0.632)	Day 14 vs Day 1	-0.495 (-0.802, 0.289)	0.149
	Day 28	0.642 (0.297, 1.384)	Day 28 vs Day 1	0.242 (-0.462, 1.868)	0.604

*: P-values were based on T-test from a linear mixed model.

Table S3B Estimated mean and relative change in CRP levels from the per-protocol analysis.

Treatment	Visit	Estimated mean (95% CI)	Time	Relative change (95% CI)	P-value*
Famotidine	Day 1	1.290 (0.672, 2.476)			
	Day 7	1.246 (0.577, 2.692)	Day 7 vs Day 1	-0.035 (-0.601, 1.336)	0.936
	Day 14	0.633 (0.324, 1.238)	Day 14 vs Day 1	-0.509 (-0.782, 0.106)	0.084
	Day 28	0.691 (0.344, 1.389)	Day 28 vs Day 1	-0.464 (-0.766, 0.226)	0.135
Placebo	Day 1	0.508 (0.287, 0.902)			
	Day 7	0.982 (0.473, 2.036)	Day 7 vs Day 1	0.932 (-0.165, 3.469)	0.120
	Day 14	0.272 (0.108, 0.684)	Day 14 vs Day 1	-0.465 (-0.801, 0.437)	0.207
	Day 28	0.667 (0.299, 1.490)	Day 28 vs Day 1	0.313 (-0.459, 2.185)	0.537

*: P-values were based on T-test from a linear mixed model.

Table S4. Estimated mean and relative change in ferritin levels from the per-protocol analysis.

Treatment	Visit	Estimated mean (95% CI)	Time	Relative change (95% CI)	P-value*
Famotidine	Day 1	143.8 (95.2, 217.0)			
	Day 7	168.3 (111.3, 254.6)	Day 7 vs Day 1	0.17 (0.01, 0.36)	0.039
	Day 14	142.2 (93.8, 215.6)	Day 14 vs Day 1	-0.01 (-0.15, 0.16)	0.892
	Day 28	103.1 (68.2, 155.8)	Day 28 vs Day 1	-0.28 (-0.38, -0.17)	<.0001
Placebo	Day 1	138.9 (98.0, 196.9)			
	Day 7	168.0 (118.3, 238.6)	Day 7 vs Day 1	0.21 (0.06, 0.39)	0.007
	Day 14	127.0 (89.1, 180.9)	Day 14 vs Day 1	-0.09 (-0.21, 0.05)	0.215
	Day 28	91.6 (64.1, 130.8)	Day 28 vs Day 1	-0.34 (-0.43, -0.24)	<.0001

*: P-values were based on T-test from a linear mixed model.

Brennan CM, *et al. Gut* 2022; 71:879–888. doi: 10.1136/gutjnl-2022-326952

Table S5. List of type-I interferon-related genes used in ssGSEA analysis.

Gene Name	Entrez Gene ID	Description
ABCE1	6059	ATP binding cassette subfamily E member 1 [Source:HGNC Symbol;Acc:HGNC:69]
ADAR	103	adenosine deaminase RNA specific [Source:HGNC Symbol;Acc:HGNC:225]
BST2	684	bone marrow stromal cell antigen 2 [Source:HGNC Symbol;Acc:HGNC:1119]
GBP2	2634	guanylate binding protein 2 [Source:HGNC Symbol;Acc:HGNC:4183]
HLA-A	3105	major histocompatibility complex, class I, A [Source:HGNC Symbol;Acc:HGNC:4931]
HLA-B	3106	major histocompatibility complex, class I, B [Source:HGNC Symbol;Acc:HGNC:4932]
HLA-C	3107	major histocompatibility complex, class I, C [Source:HGNC Symbol;Acc:HGNC:4933]
IFI35	3430	interferon induced protein 35 [Source:HGNC Symbol;Acc:HGNC:5399]
IFI6	2537	interferon alpha inducible protein 6 [Source:HGNC Symbol;Acc:HGNC:4054]
IFIT1	3434	interferon induced protein with tetratricopeptide repeats 1 [Source:HGNC Symbol;Acc:HGNC:5407]
IFIT2	3433	interferon induced protein with tetratricopeptide repeats 2 [Source:HGNC Symbol;Acc:HGNC:5409]
IFIT3	3437	interferon induced protein with tetratricopeptide repeats 3 [Source:HGNC Symbol;Acc:HGNC:5411]
IFITM2	10581	interferon induced transmembrane protein 2 [Source:HGNC Symbol;Acc:HGNC:5413]
IRF2	3660	interferon regulatory factor 2 [Source:HGNC Symbol;Acc:HGNC:6117]
IRF3	3661	interferon regulatory factor 3 [Source:HGNC Symbol;Acc:HGNC:6118]
IRF6	3664	interferon regulatory factor 6 [Source:HGNC Symbol;Acc:HGNC:6121]
IRF9	10379	interferon regulatory factor 9 [Source:HGNC Symbol;Acc:HGNC:6131]
ISG15	9636	ISG15 ubiquitin like modifier [Source:HGNC Symbol;Acc:HGNC:4053]
ISG20	3669	interferon stimulated exonuclease gene 20 [Source:HGNC Symbol;Acc:HGNC:6130]
MX1	4599	MX dynamin like GTPase 1 [Source:HGNC Symbol;Acc:HGNC:7532]
MX2	4600	MX dynamin like GTPase 2 [Source:HGNC Symbol;Acc:HGNC:7533]
OAS1	4938	2'-5'-oligoadenylate synthetase 1 [Source:HGNC Symbol;Acc:HGNC:8086]
OAS2	4939	2'-5'-oligoadenylate synthetase 2 [Source:HGNC Symbol;Acc:HGNC:8087]
OAS3	4940	2'-5'-oligoadenylate synthetase 3 [Source:HGNC Symbol;Acc:HGNC:8088]
OASL	8638	2'-5'-oligoadenylate synthetase like [Source:HGNC Symbol;Acc:HGNC:8090]
PSMB8	5696	proteasome 20S subunit beta 8 [Source:HGNC Symbol;Acc:HGNC:9545]
RNASEL	6041	ribonuclease L [Source:HGNC Symbol;Acc:HGNC:10050]

Brennan CM, *et al. Gut* 2022; 71:879–888. doi: 10.1136/gutjnl-2022-326952

RSAD2	91543	radical S-adenosyl methionine domain containing 2 [Source:HGNC Symbol;Acc:HGNC:30908]
SAMHD1	25939	SAM and HD domain containing deoxynucleoside triphosphate triphosphohydrolase 1 [Source:HGNC Symbol;Acc:HGNC:15925]

Brennan CM, *et al. Gut* 2022; 71:879–888. doi: 10.1136/gutjnl-2022-326952
